# Exosomes Mediate APP Dysregulation *via* APP-miR-185-5p Axis

**DOI:** 10.3389/fcell.2022.793388

**Published:** 2022-02-11

**Authors:** Lu Ding, Xiaoyu Yang, Xiaohuan Xia, Yunxia Li, Yi Wang, Chunhong Li, Yiyan Sun, Ge Gao, Shu Zhao, Shiyang Sheng, Jianhui Liu, Jialin C. Zheng

**Affiliations:** ^1^ Department of Anesthesiology, Tongji Hospital Affiliated to Tongji University School of Medicine, Shanghai, China; ^2^ Center for Translational Neurodegeneration and Regenerative Therapy, Tongji Hospital Affiliated to Tongji University School of Medicine, Shanghai, China; ^3^ Center for Translational Neurodegeneration and Regenerative Therapy, Shanghai 10th People’s Hospital Affiliated to Tongji University School of Medicine, Shanghai, China; ^4^ Translational Research Institute of Brain and Brain-Like Intelligence, Shanghai Fourth People’s Hospital Affiliated to Tongji University School of Medicine, Shanghai, China; ^5^ Collaborative Innovation Center for Brain Science, Tongji University, Shanghai, China

**Keywords:** exosome, miR-185-5p, APP, Alzheimer’s disease, brain, extracellular vesicle, microRNA, serum

## Abstract

APP misexpression plays a crucial role in triggering a complex pathological cascade, leading to Alzheimer’s disease (AD). But how the expression of APP is regulated in pathological conditions remains poorly understood. In this study, we found that the exosomes isolated from AD mouse brain promoted APP expression in neuronal N2a cells. Moreover, exosomes derived from N2a cells with ectopic expression of APP (APP-EXO) also induced APP dysregulation in normal N2a cells. Surprisingly, the effects of APP-EXO on APP expression in recipient cells were not mediated by the direct transferring of *APP* gene products. Instead, the effects of APP-EXO were highly likely mediated by the reduction of the expression levels of exosomal miR-185-5p. We found that the 3′UTR of *APP* transcripts binds to miR-185-5p, therefore inhibiting the sorting of miR-185-5p to exosomes. N2a cell-derived exosomes with less amount of miR-185-5p exert similar roles in APP expression to APP-EXO. Lastly, we demonstrated a significant decline of serum exosomal miR-185-5p in AD patients and AD mice, versus the corresponding controls. Together, our results demonstrate a novel mechanism in the exosome-dependent regulation of APP, implying exosomes and exosomal miRNAs as potential therapeutic targets and biomarkers for AD treatment and diagnosis, respectively.

## Introduction

In recent decades, the neurobiology of aging has expanded greatly as a scientific discipline. The abnormal age-related changes in the central nervous system (CNS) have been considered as one key determinant in the pathogenesis of various neurodegenerative diseases, such as Alzheimer’s disease (AD), Parkinson’s disease (PD), and glaucoma (Jucker and Ingram, 1997; Kalia and Lang, 2015; Mancino et al., 2018). AD is the most common age-related neurodegenerative disease worldwide and the number one cause for dementia in the aged population (Danborg et al., 2014; Reitz and Mayeux, 2014). One key pathological characteristic of AD is the accumulation of insoluble forms of amyloid-β (Aβ) in plaques in extracellular spaces (Masters et al., 2015). In pathological conditions, amyloid precursor protein (APP) is proteolyzed by β- and γ-secretase, generating Aβ peptides of 40 or 42 amino acids (Aβ_40_ or Aβ_42_) (Selkoe, 2001). Both peptides, especially Aβ_42_, are hydrophobic and tend to fold in β-sheet structure which recruits other extracellular proteins to form senile plaques (Masters et al., 2015). The classical Aβ hypothesis of AD claims that the mutant or misexpression of APP generates excessive Aβ (Hardy and Selkoe, 2002), leading to neurotoxicity (O'Brien and Wong, 2011), neuroinflammation (Cai et al., 2014), and regeneration deficiency (Scopa et al., 2019). Due to the key roles of APP in the pathogenesis of AD, APP overexpression has been widely applied in the establishment of AD models *in vivo* (Webster et al., 2014; Gao et al., 2019) and *in vitro* (Atluri et al., 2019; Lin et al., 2019). Although APP has been considered as one of the most important risk genes for AD, it remains largely unknown how the expression of APP is regulated under pathological conditions.

Emerging evidence implicates exosomes as a key microenvironment element that contributes to the progression of AD. Exosomes are small bilipid layer-enclosed extracellular vesicles (30–200 nm) that are released by virtually all CNS cells (Xia et al., 2019b). Exosomes have been reported to participate in the spreading of Aβ in the brain (Dinkins et al., 2014). The inhibition of exosome release halts the propagation of tau, an AD-related protein whose phosphorylation and cleavage are induced by Aβ (Hernandez and Avila, 2008; Asai et al., 2015). The functions of exosomes are mediated by their contents such as miRNAs (Xia et al., 2019b). Mounting studies have demonstrated distinct profiles of exosomal miRNAs in various biological fluids collected from AD patients, compared with healthy donors (Liu et al., 2014; Lugli et al., 2015; Yang et al., 2018). Currently, multiple miRNAs including miR-455-3p, miR-193b-3p, and miR-31-5p have been reported to target *APP* 3′ untranslated region (UTR) and inhibit the expression of APP protein *in vivo* and *in vitro* (Liu et al., 2014; Kumar et al., 2019; Barros-Viegas et al., 2020; Kumar et al., 2021; Kumar and Reddy, 2021). Hence, aforementioned literatures suggest the importance to investigate the roles of exosomes, especially exosomal miRNAs, in the pathogenesis of AD. In this study, we firstly demonstrated that exosomes isolated from the AD mouse brains (AD-EXO) significantly elevated APP expression levels in recipient cells. We next collected exosomes from conditioned medium of N2a cells with APP overexpression (APP-N2A), an *in vitro* AD model, and observed that APP-N2A-derived exosomes (APP-EXO) also enhanced APP expression in recipient cells. Interestingly, the effects of exosomes on APP dysregulation were mediated by reducing the levels of exosomal miR-185-5p, an inhibitory microRNA (miRNA) for APP expression, instead of the direct transferring of *APP* gene products to recipient cells. We further demonstrated that APP in producer cells regulated the sorting of miR-185-5p to exosomes *via* the direct interaction of *AP*P 3′UTR and miR-185-5p. At last, we observed a significant reduction of miR-185-5p levels in exosomes isolated from the AD patients and mice sera, compared with the corresponding controls. Together, our study discovered a novel mechanism for APP expression regulation *via* exosome-mediated delivery of miR-185-5p, indicating the important roles of exosomes and their contents in the pathogenesis of AD.

## Materials and Methods

### Culture of N2a Cell Line

N2a neuronal cells were purchased from the Chinese Academy of Sciences Cell Bank, and cultured in DMEM (Gibco) containing 10% FBS (Gibco) and 100 U/ml penicillin and streptomycin (ThermoFisher).

### Mice

APP/PS1 mice and C57 mice (purchased from Shanghai Model Organisms Center) are housed and bred in the Comparative Medicine animal facilities of Tongji University School of Medicine (TUSM). All procedures were conducted according to protocols approved by the Institutional Animal Care and Use Committee of TUSM. Mouse genotype was validated by PCR.

### Study Population

The design of the present study was approved by the ethics committee of Tongji Hospital affiliated to Tongji University School of Medicine (Shanghai, China), and the written informed consents were obtained from all participants. A total of three AD patients (males, mean age 67.3 ± 4.7) and three healthy donors (males, mean age 70.0 ± 7.1) were selected for this study. Peripheral blood was collected from each patient after fasting for 12 h. The serum was separated by centrifugation at 3,000 *g* for 10 min in room temperature, followed by centrifugation at 12,000 *g* for 5 min at 4°C.

### Collection of Exosomes in Conditioned Medium, Brain Extracellular Spaces, and Serum

Exosomes were isolated from the culture medium of N2a cells as previously described (Ma et al., 2018). Briefly, 3.5 × 10^6^ N2a cells were culture in 10 cm dish. 24 h before collecting supernatants, the culture medium was replaced with FBS free medium containing DMEM (Gibco) and 100 U/ml penicillin and streptomycin (ThermoFisher) only. After conditioned media collection, exosomes were isolated by sequential centrifugation: conditioned media was first centrifuged at 300 g for 10 min to eliminate flowing cells, at 3,000 g for 20 min to eliminate cellular debris, at 10,000 g for 30 min to eliminate intracellular organelles and then at 100,000 g for 2 h to precipitate exosomes. All steps of centrifugation were handled at 4°C. For brain exosome isolation, fresh mouse brains were dissected and treated with 20 units/ml papain (Worthington) in Hibernate E solution (5 ml/brain) for 30 min at 37°C. The brain tissue was gently homogenized in 10 ml cold Hibernate E solution. The brain homogenate was sequentially filtered through a 40 μm mesh filter (BD Biosciences) and a 0.2 μm syringe filter (Thermo Scientific). Exosomes in the filtrate were collected through gradient centrifugation as described above. Exosomes in the serum were isolated using ExoQuick Exosome Precipitation kit (System Biosciences). Exosomes were resuspended in PBS and stored in −80°C until required.

### Nanoparticle Tracking Analysis

Nanoparticle Tracking Analysis (NTA) was carried out as previously described (Ma et al., 2019). Briefly, 1 ml of exosome suspension was used for NanoSight analysis. NTA was assessed on NanoSight NS300 system (Malvern Instruments, UK) with a sCMOS camera. The conditions of the measurements were set at 25°C, 1 cP viscosity, 25 s per capture frame, and 60 s measurement time. Three individual measurements were applied for measuring the sizes of exosomes.

### Transmission Electron Microscopy

Transmission Electron Microscop (TEM) was carried out as previously described (Ma et al., 2019). Briefly, purified exosomes were negatively stained and then spread on the copper grids. The droplets of exosomes were removed with filter paper and air-dried at room temperature. Images were obtained using transmission electron microscopy (JEM-1230, JEOL Ltd.).

### Human APP Full-Length/CDS/3′UTR Plasmids, miR-185-5p Mimics/Inhibitor, and Transfection

The plasmids for the ectopic expression of human *APP* full-length, *APP* CDS, and *APP* 3′UTR sequences were constructed by Generay (Generay Biotech Co., Ltd., Shanghai). Plasmid backbone pcDNA3.1 was purchased from Generay. APP transcript sequences were obtained from UCSC Genome Browser (http://genome.ucsc.edu/) and inserted into pcDNA3.1 using restriction enzymes NheI and NotI for genomic digestion. The mimics control, miR-185-5p mimics, inhibitor control, and miR-185-5p inhibitor were purchased from GenePharma (GenePharma Co., Ltd., Shanghai). Transfection was performed using the Lipofectamine 2000 reagent (Invitrogen) according to the manufacturer’s instruction.

### Quantitative Reverse Transcription Polymerase Chain Reaction (RT-qPCR)

The messenger RNA (mRNA) and miRNA were isolated from cell samples using RNeasy mini kit (Qiagen) according to the manufacturer’s instructions. Genomic DNA was removed and cDNA was synthesized using DNase I digestion kit (Qiagen) and miScript II RT kit (Qiagen), respectively. Transcripts were amplified using specific primer sets (Supplementary Table S1) and SYBR green PCR kit (Qiagen) with the ABI7500 (Applied Biosystems). Reactions were run in triplicates for each sample and no-template blanks were used as negative controls. Values were normalized to the *Gapdh* (for mRNA) and U6 snRNA (for miRNA).

### Western Blotting

Western blotting was performed as previously described (Gao et al., 2019). Exosomes or cells were lysed in RIPA lysis and extraction buffer (ThermoFisher) containing a protease inhibitor cocktail (Sigma). Protein concentrations were determined with a BCA Protein Assay Kit (Pierce). Proteins (20–30 mg) from exosome and cell lysates were separated by sodium dodecyl sulfate polyacrylamide gel electrophoresis (SDS-PAGE) and electrophoretic transferred to polyvinylidene fluoride membranes (Millipore and Bio-Rad). Membranes were incubated with primary antibodies for APP (rabbit, Abcam, 1:1,000), CD9 (rabbit, Abcam, 1:2000), Flottlin2 (mouse, BD Biosciences; 1:5,000), overnight at 4°C followed by a secondary anti-rabbit or anti-mouse antibody (Cell Signaling Technologies, 1:10,000) incubation. Antigen-antibody complexes were visualized by Pierce ECL Western Blotting Substrate (ThermoFisher). For data quantification, films were scanned with a CanonScan 9950F scanner; the acquired images were analyzed using ImageJ program.

### Small RNA Sequencing and Bioinformatics Analysis

Total RNA was extracted from N2a cell-derived exosomes and 3 μg total RNA per sample was used as input material for the small RNA library. Sequencing libraries were generated using NEBNext® Multiplex Small RNA Library Prep Set for Illumina® (NEB). The clustering of the index-coded samples was performed on a cBot Cluster Generation System using TruSeq SR Cluster Kit v3-cBot-HS (Illumia). After cluster generation, the library preparations were sequenced on an Illumina Hiseq 2,500/2000 platform and 50 bp single-end reads were generated. Raw data (raw reads) of fastq format were firstly processed through custom perl and python scripts for quality control. The small RNA tags were mapped to reference sequence by Bowtie without mismatch to analyze their expression and distribution on the reference. Mapped small RNA tags were used to looking for known miRNA. miRBase 20.0 was used as reference for known miRNA, miRDeep2 and sRNA-tools-cli were used to obtain novel miRNAs and draw the secondary structures, respectively. miRNA expression levels were estimated by TPM (transcript per million) and differentially expressed miRNAs between two groups were determined (*p* ≤ 0.05). The targets of differentially expressed miRNAs were predicted using TargetScan and miRanda databases according to the scoring criteria of each software. The target genes whose context score percentage is less than 50 or max energy is greater than −10 were removed in targetscan algorithm and Miranda algorithm, respectively. The intersection of predicted targets of both databases was taken as the final target genes of differential expressed miRNAs. Predicted targets of differentially expressed miRNAs were mapped to Gene ontology (GO) and Kyoto Encyclopedia of Genes and Genomes (KEGG) pathways analysis. GO and KEGG enrichment analysis was performed using The Database for Annotation, Visualization and Integrated Discovery (DAVID) (http://david.ncifcrf.gov/).

### Dual-Luciferase Reporter Assay

Dual-luciferase reporter assay was performed as previously described (Xia et al., 2019a). The APP wild type 3′UTR (APP WT 3′UTR) and miR-185-5p binding site-mutated 3′UTR (APP MU 3′UTR) were synthesized by Genewiz (Genewiz, Suzhou, China) and cloned into the PmeI and SacI site of the pmirGLO vector (Promega, Beijing, China), downstream of the firefly luciferase gene. For the luciferase assay, 3 × 10^4^ 293T cells were cultured in 96-well plates with DMEM (Gibco) containing 10% FBS (Gibco) and 100 U/ml penicillin and streptomycin (ThermoFisher). Cells were co-transfected with miR-185-5p mimics and either APP WT 3′UTR or APP MU 3′UTR dual-luciferase reporter vector after reaching ∼70% confluency. Serum-free Opti-MEM (ThermoFisher) was used to prepare the transfection solution, and Lipofectamine 2000 reagent (Invitrogen) was used for transfection. 24 h post transfection, Dual-Luciferase® Reporter Assay System (Promega) was used to determine the luciferase activities on SpectraMax M5 microplate readers (Molecular Devices). The activity of Renilla luciferase was used to normalize that of firefly luciferase.

### Statistical Analyses

All results are the means of at least three independent experiments ± s. d. Data from two groups were evaluated statistically by two-tailed, paired or unpaired student *t* test. Data from multiple groups were evaluated statistically by one-way ANOVA followed by Tukey’s post hoc test. Significance was considered when *p* < 0.05.

## Results

### Exosomes From AD Mouse Brains Induce APP Expression in Neuronal N2a Cells

**FIGURE 1:**
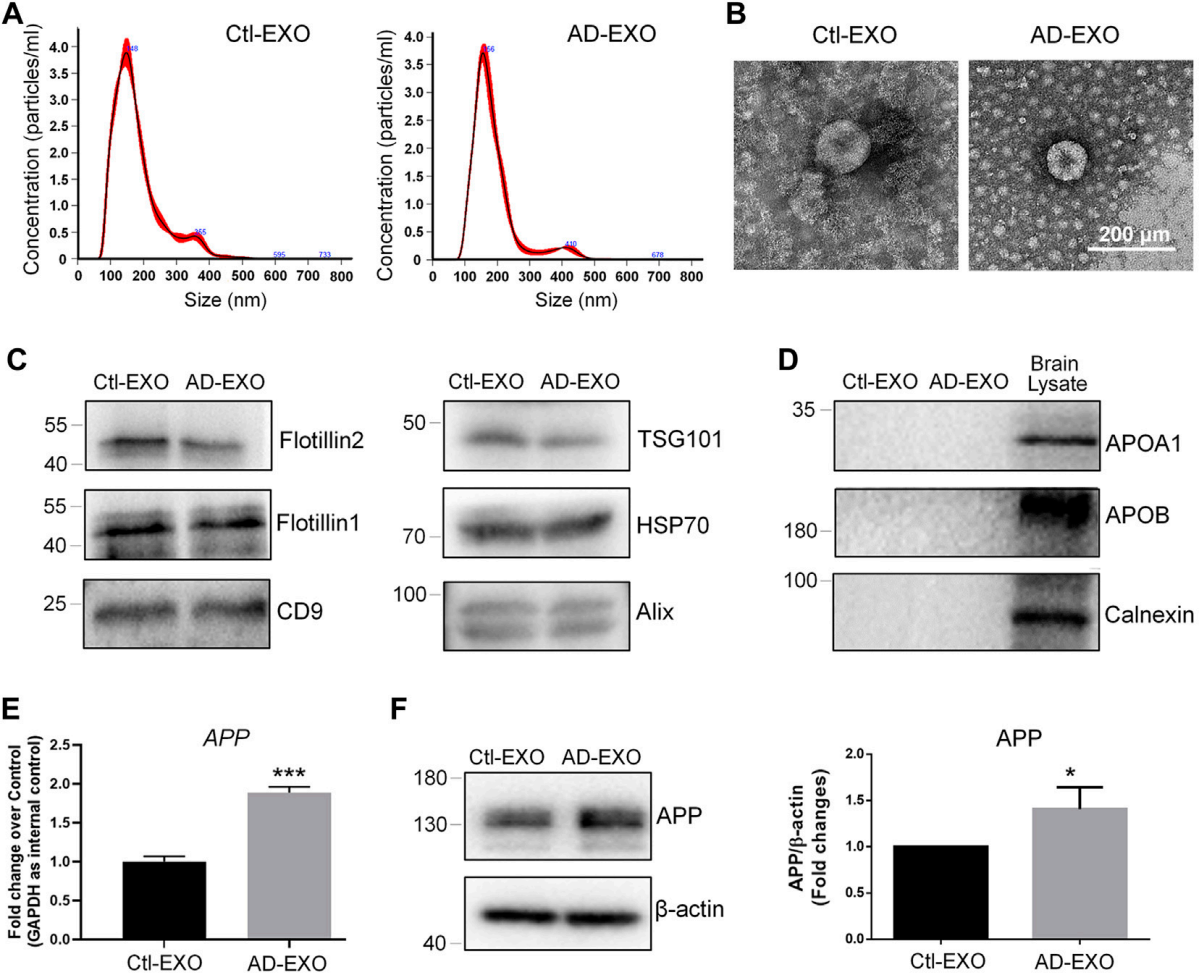
AD-EXO promote APP expression in N2a cells. **(A)** Particle-size distribution and concentration of exosomes were determined by NTA. **(B)** Ctl-EXO and AD-EXO were visualized under TEM using negative staining. **(C)** The expression levels of exosome positive markers Flotillin2, Flotillin1, CD9, TSG101, HSP70, and CD9 in protein lysates of exosomes were determined by western blotting. **(D)** The expression levels of exosome negative markers Apoa1, Apob, and Calnexin in protein lysates of exosomes were determined by western blotting. **(E)** The *APP* transcript levels in N2a cells co-cultured with either AD-EXO or Ctl-EXO were determined by RT-qPCR. **(F)** The APP protein levels in N2a cells co-cultured with either AD-EXO or Ctl-EXO were determined by western blotting. Data were represented as mean ± s. d. from three independent experiments. * and *** denote *p* < 0.05 and *p* < 0.001, respectively.

To investigate the effects of exosomes on APP expression, we first isolated exosomes from cortical and hippocampal tissues of 7-month-old APP/PS1 mice (AD-EXO) and control C57 mice (Ctl-EXO). The purity of exosomes was examined by NTA and western blotting. NTA results suggested that the sizes of AD-EXO and Ctl-EXO were between 100 and 200 nm, matching with the typical size distribution of exosomes (Figure 1A). The morphology of exosomes was visualized *via* TEM, which showed distinct cup-shaped structure of exosomes (Figure 1B). Western blotting detected strong expression of exosome positive markers, Flotillin2, Flotillin1, CD9, TSG101, HSP70, and CD9, in both AD-EXO and Ctl-EXO (Figure 1C). Moreover, the negative markers of exosomes, Apoa1, Apob, and Calnexin, were absent in both AD-EXO and Ctl-EXO, further confirming the purity of exosomes (Figure 1D). To determine the effects of exosomes on APP expression, we co-cultured N2a cells with either 20 μg/ml AD-EXO or Ctl-EXO for 2 days. RT-qPCR analysis showed higher levels of *APP* transcripts in AD-EXO-treated group, compared with Ctl-EXO group (Figure 1E). Western blotting further revealed a significant increase of the expression levels of APP proteins in AD-EXO-treated cells versus Ctl-EXO group (Figure 1F). Thus, our results suggest an important role of exosomes from AD mouse brains in APP dysregulation in neuronal cells.

### Exosomes Derived From N2a Cells With APP-Overexpression Promote APP Expression in Recipient Cells

**FIGURE 2:**
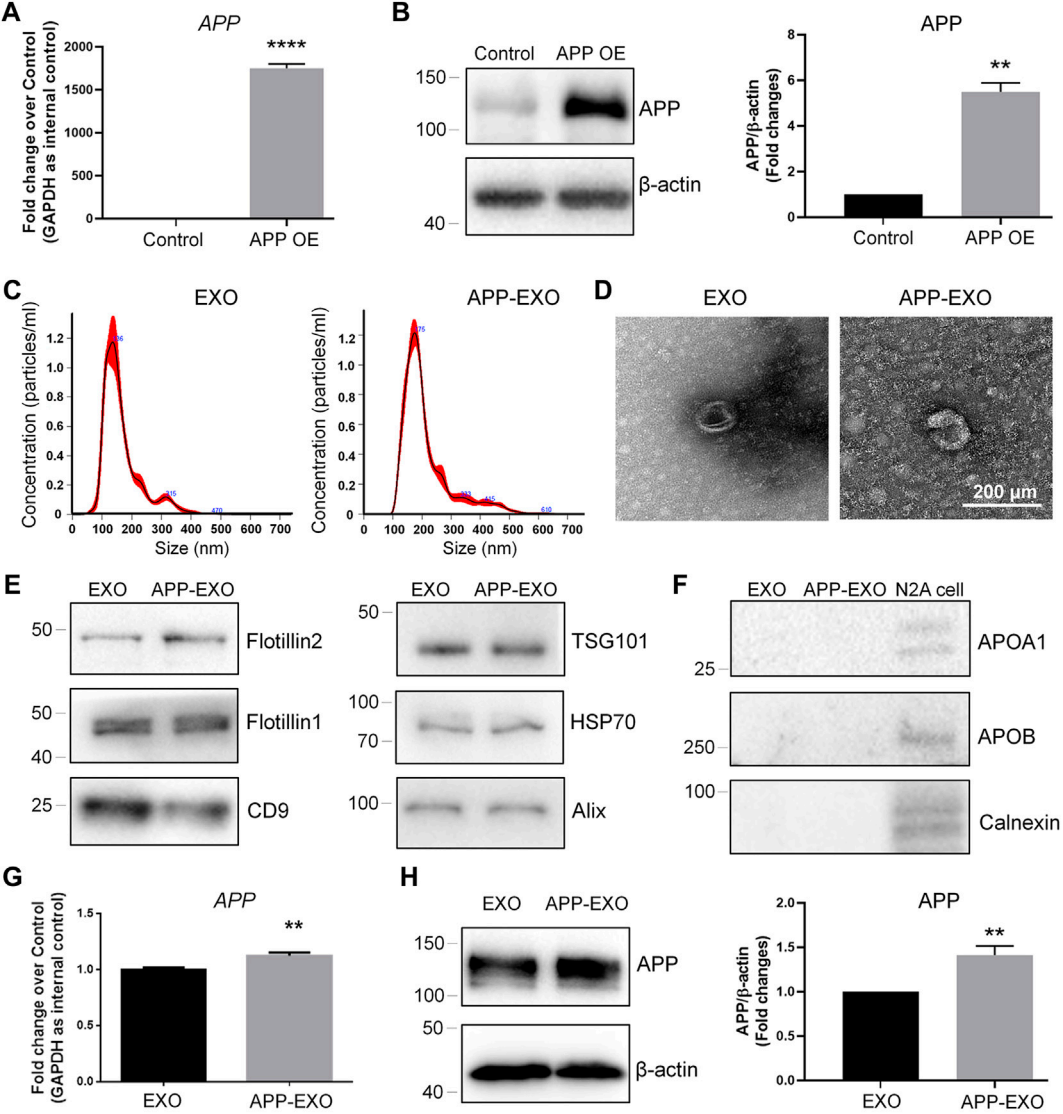
APP-EXO promote APP expression in recipient cells. **(A,B)** N2a cells were transfected with either control vector or APP OE plasmid for 2 days. The expression levels of *APP* transcripts and APP proteins were determined by RT-qPCR **(A)** and western blotting **(B)**, respectively. **(C)** Particle-size distribution and concentration of exosomes were determined by NTA. **(D)** EXO and APP-EXO were visualized under TEM using negative staining. **(E)** The expression levels of exosome positive markers Flotillin2, Flotillin1, CD9, TSG101, HSP70, and CD9 in protein lysates of exosomes were determined by western blotting. **(F)** The expression levels of exosome negative markers Apoa1, Apob, and Calnexin in protein lysates of exosomes were determined by western blotting. **(G)** The *APP* transcript levels in N2a cells co-cultured with APP-EXO or EXO were determined by RT-qPCR. **(H)** The APP protein levels in N2a cells co-cultured either APP-EXO or EXO were determined by western blotting. Data were represented as mean ± s. d. from three independent experiments. ** and **** denote *p* < 0.01 and *p* < 0.0001, respectively.

To further confirm the exosome-mediated APP dysregulation and investigate the underlying mechanisms, we established an *in vitro* AD model by ectopically expressing full-length *APP* mRNAs in N2a cells to mimic the pathological conditions of AD *in vivo*. The overexpression of *APP* transcripts and APP proteins in N2a was validated by RT-qPCR (Figure 2A) and western blotting (Figure 2B), respectively. APP-overexpressed (APP OE) cell-derived exosomes (APP-EXO) or empty vector-transfected (control) cell-derived exosomes (EXO) were then collected, visualized, and validated *via* NTA, TEM, and western blotting assays (Figure 2C–F). To determine the effects of APP-EXO on APP expression, we co-cultured N2a cells with either 20 μg/ml APP-EXO or EXO for 2 days. Both RT-qPCR and western blotting results revealed a significant increase of the expression levels of *APP* transcripts (Figure 2G) and APP proteins (Figure 2H) in APP-EXO-treated cells versus EXO group, indicating the positive effects of APP-EXO on APP expression in neuronal cells.

### Exosomes Promote APP Expression Independent to the Direct Transferring of *APP* Gene Products

**FIGURE 3:**
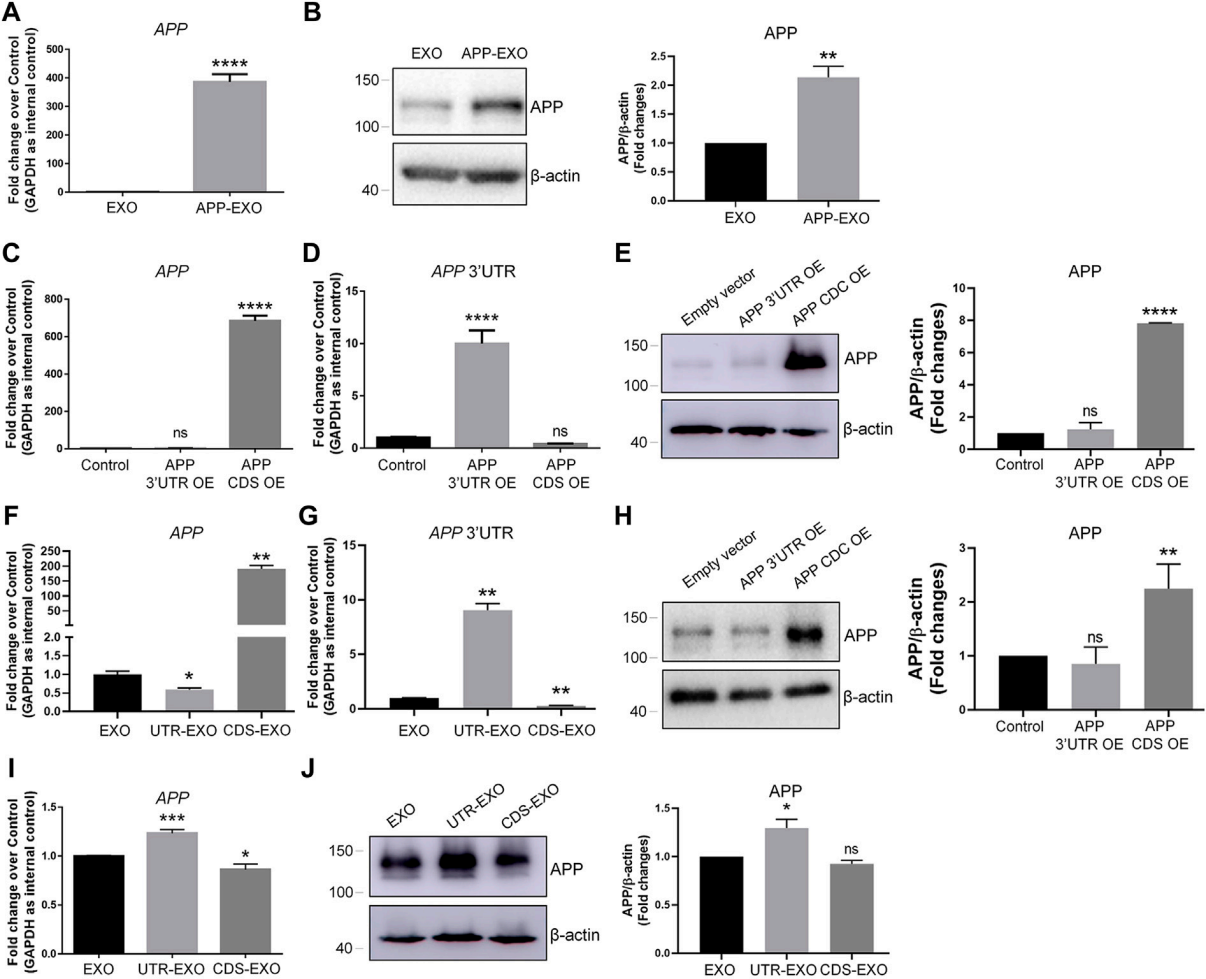
APP-EXO promote APP expression in recipient cells independent to the direct delivery of *APP* gene products. **(A,B)** The expression levels of *APP* transcripts and APP proteins in exosomes were determined by RT-qPCR **(A)** and western blotting **(B)**, respectively. **(C,D)** N2a cells were transfected with empty control vector, APP 3′UTR OE plasmid, or APP CDS OE plasmid for 2 days. The expression levels of *APP* transcripts **(C)** and *AP*P 3′UTR **(D)** were determined by RT-qPCR. **(E)** The APP protein levels in control, APP 3′UTR OE, and APP CDS OE groups were determined by western blotting. **(F,G)** The expression levels of *APP* transcripts **(F)** and *APP* 3′UTR **(G)** in EXO, UTR-EXO, and CDS-EXO were determined by RT-qPCR. **(H,I)** N2a cells were co-culture with EXO, UTR-EXO, or CDS-EXO for 2 days. The expression levels of *APP* transcripts **(H)** and APP proteins **(I)** were determined by RT-qPCR and western blotting, respectively. Data were represented as mean ± s. d. from three independent experiments. *, **, and **** denote *p* < 0.05, *p* < 0.01, and *p* < 0.0001, respectively. ns denotes no significance.

We next investigated the possible mechanisms of exosome-induced APP dysregulation. The simplest and most obvious one is that exosomes may directly transfer *APP* transcripts and APP proteins to target cells (Perez-Gonzalez et al., 2012). To test this premise, we first compared the expression levels of *APP* transcripts and APP proteins in APP-EXO and EXO. Both RT-qPCR and western blotting results indicated that APP-EXO contains higher levels of *APP* transcripts (Figure 3A) and APP proteins (Figure 3B), compared with EXO. To further confirm our observations, we then transfected N2a cells with plasmid that overexpresses the coding DNA sequence (CDS) region of *APP* transcript. The empty (control) plasmid and plasmid containing the 3′ UTR of *APP* transcript were used as controls. Both RT-qPCR and western blotting results suggested a significant up-regulation of *APP* CDS and APP proteins in *APP* CDS-overexpressed (CDS OE) N2a cells, compared with *AP*P 3′UTR-overexpressed (3′UTR OE) and control cells (Figures 3C,E). Moreover, APP 3′UTR levels were elevated only in 3′UTR OE group versus CDS OE and control groups (Figure 3D). Our observations suggested that CDS OE has similar promotional effects to APP OE on the expression of *APP* transcripts and proteins in N2a cells. Based on the results, we proposed that exosomes derived from CDS OE cells (CDS-EXO) should have also similar promotional effects to APP-EXO on the expression of *APP* transcripts and proteins in recipient cells if exosomes regulated APP expression through the direct transferring of *APP* gene products among cells.

We then collected CDS-EXO and exosomes derived from 3′UTR OE cells (UTR-EXO). The sorting of *APP* gene products into exosomes was confirmed by RT-qPCR and western blotting. The results showed that *APP* CDS expression levels were significantly higher in CDS-EXO, but not in UTR-EXO, compared with EXO (Figure 3F). *APP* 3′UTR levels were significantly higher in UTR-EXO, compared with both CDS-EXO and EXO (Figure 3G). Moreover, APP protein levels were significantly elevated in CDS-EXO, but not in UTR-EXO, compared with EXO, confirming the enrichment of *APP* gene products in CDS-EXO (Figure 3H). Afterwards, we co-cultured N2a cells with either CDS-EXO, UTR-EXO, or EXO for 2 days. Surprisingly, both RT-qPCR and western blotting results showed that CDS-EXO failed to elevate the expression levels of *APP* transcripts and APP proteins, respectively (Figures 3I,J). In contrast, UTR-EXO significantly increased the expression of *APP* transcripts and APP proteins in co-cultured cells (Figures 3I,J). Therefore, our results suggested that APP-EXO regulated APP expression in recipient cells independent to the direct transferring of *APP* gene products.

### Exosomes Derived From Neuronal Cells With APP-Overexpression Exhibit Distinct miRNA Profiles

**FIGURE 4:**
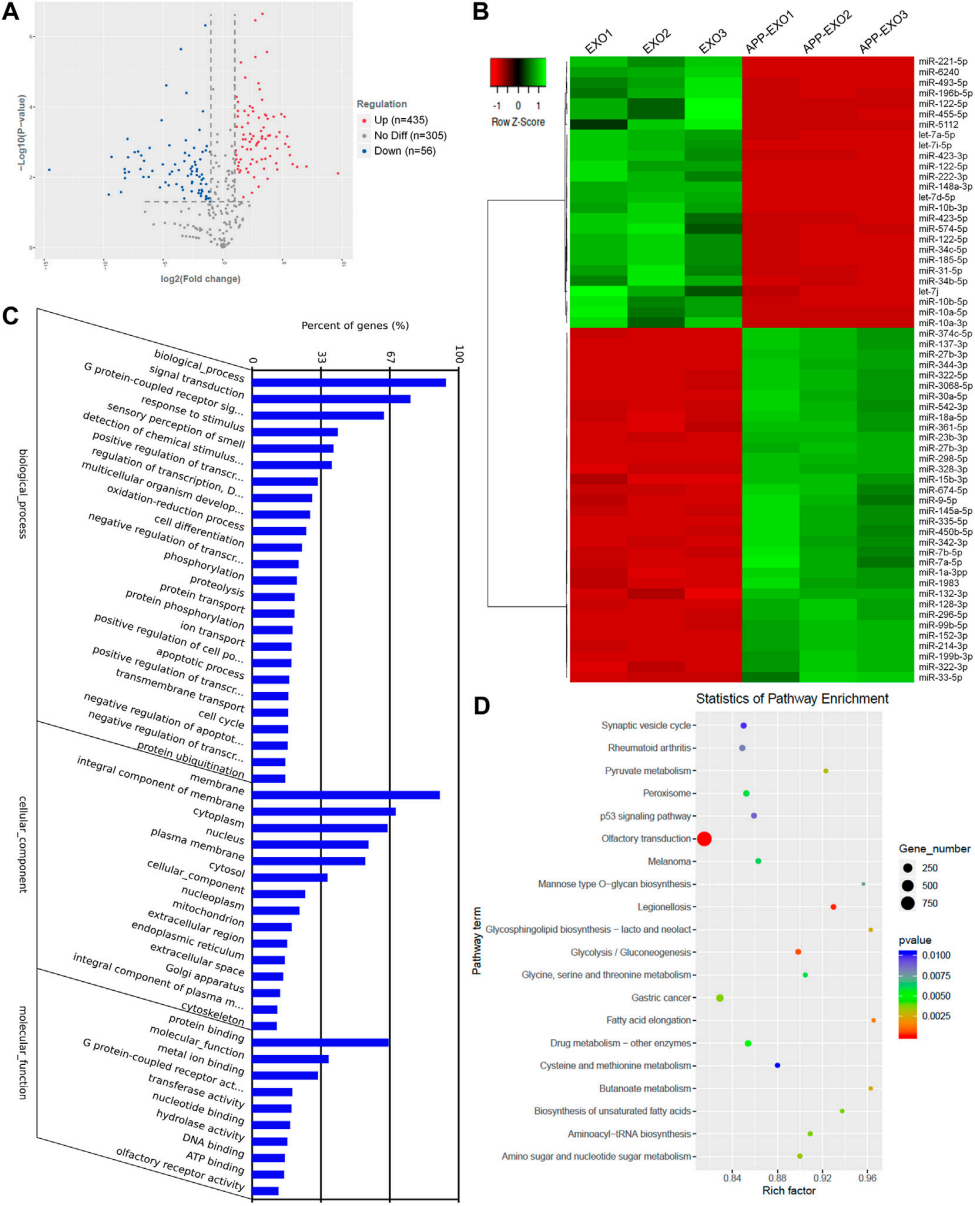
APP overexpression significantly alters exosomal miRNA profiles. **(A)** The volcano plot was generated based on the logarithm of the *p*-values and the log fold change of all detected miRNAs in APP-EXO and EXO. **(B)** The top 60 differentially expressed miRNAs between APP-EXO and EXO are represented in the heat map and hierarchical clustering-based dendrograms. **(C)** The enriched gene ontology (GO) terms in the predicted targets of differentially expressed miRNAs were determined by GO analysis. **(D)** The enriched signaling pathways in the predicted targets of differentially expressed miRNAs were determined by KEGG analysis.

To understand the mechanisms of UTR-EXO-mediated APP regulation, we first carried out small RNA sequencing to determine the miRNA signatures of APP-EXO and EXO, given the importance of mRNA 3′UTR-miRNA interaction in mediating the content sorting and functions of exosomes (Squadrito et al., 2014). 796 miRNAs were detected in either APP-EXO or EXO (Supplementary Table S2). 435 and 56 miRNAs were identified that were significantly up-regulated and down-regulated, respectively, in APP-EXO versus EXO (Figure 4A, Supplementary Table S3). The heatmap that represented the top 60 differentially expressed miRNAs also demonstrated the distinct profiles of exosomal miRNAs between two exosome populations (Figure 4B). GO analysis on biological process revealed that the predicted target genes of differentially expressed miRNAs were strongly associated with response to stimulus, cell differentiation, protein phosphorylation, and apoptotic process that might play roles in the pathogenesis of AD (Figure 4C). More importantly, KEGG analysis of the predicted target genes identified synaptic vesicle cycle, p53 pathway, Pyruvate pathway, glycolysis, and glycosphingolipid biosynthesis that were linked to AD (Figure 4D). Taken together, our study demonstrated distinct miRNA signatures in exosomes derived from N2a cells with and without APP dysregulation, implying an involvement of exosomal miRNAs in the regulation of AD-related pathogenic networks.

### *APP* 3′UTR Directly Binds to miR-185-5p and Inhibits miR-185-5p Sorting to Exosomes

**FIGURE 5:**
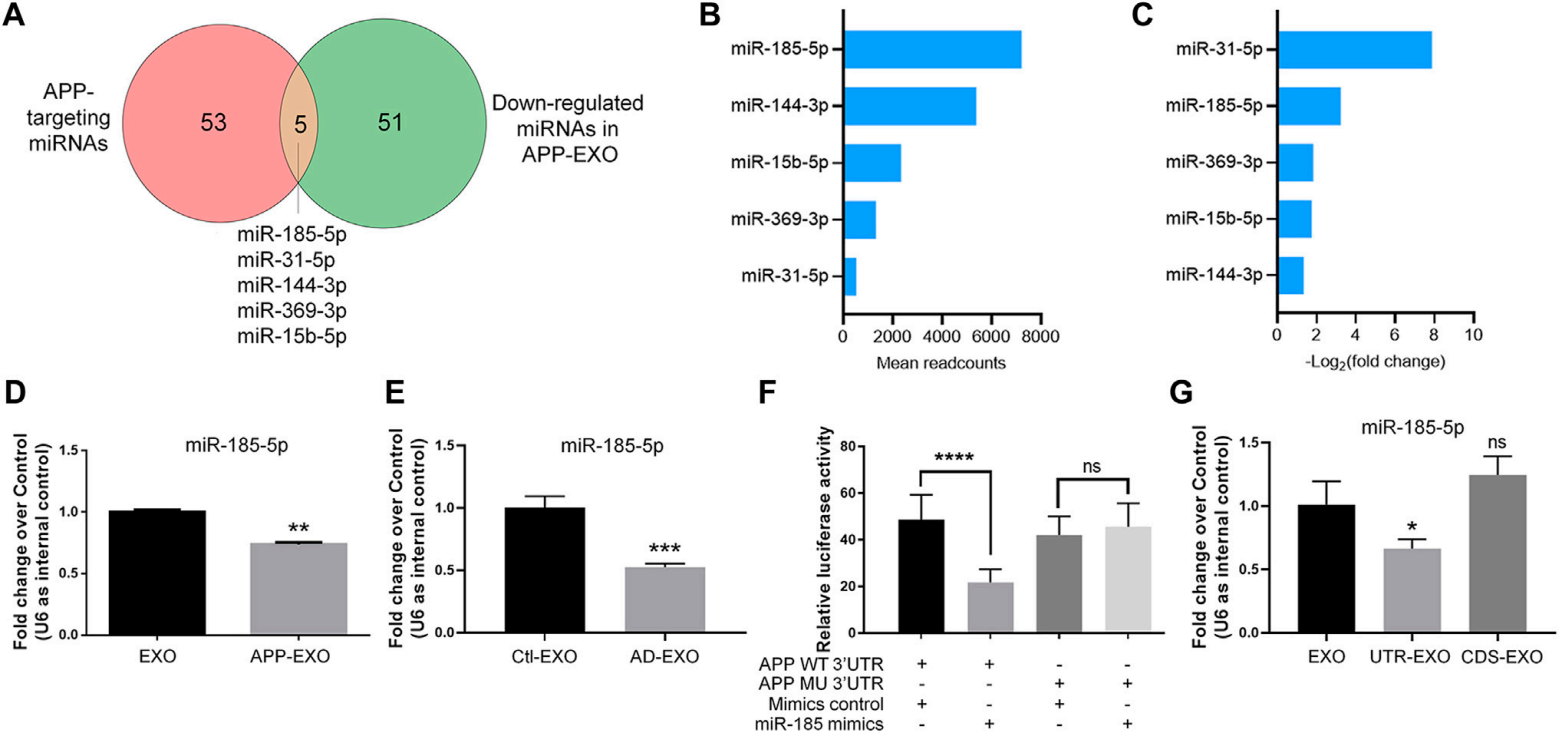
APP overexpression inhibits miR-185-5p sorting to exosomes *via* the direct interaction between APP 3′UTR and miR-185-5p. **(A)** The numbers of APP-targeting miRNAs and down-regulated miRNAs in APP-EXO were shown in the Venn diagram. The overlapping miRNAs were listed. **(B)** The readcounts of five overlapping miRNAs in EXO were ranked from more to less. **(C)** The fold changes of five overlapping miRNAs between APP-EXO and EXO were ranked from more to less. **(D)** The down-regulation of miR-185-5p in APP-EXO was determined by RT-qPCR. **(E)** The down-regulation of miR-185-5p in AD-EXO was determined by RT-qPCR. **(F)** Repression of luciferase activities by the *APP* 3′UTR were dependent on miR-185-5p. Firefly luciferase activities were normalized to the internal control, Renilla luciferase activities. **(G)** The down-regulation of miR-185-5p in UTR-EXO, but not CDS-EXO, was confirmed by RT-qPCR. Data were represented as mean ± s. d. from three independent experiments. *, **, ***, and **** denote *p* < 0.05, *p* < 0.01, *p* < 0.001, and *p* < 0.0001, respectively. ns denotes no significance.

To further identify the key miRNAs that mediate the effects of APP-EXO on APP expression in recipient cells, we identified the overlapping miRNAs (miR-185-5p, miR-31-5p, miR-144-3p, miR-369-3p, and miR-15b-5p) between the down-regulated miRNAs in APP-EXO and miRNAs that conservatively bind to *APP* 3′UTR (Figure 5A). Among these miRNAs in EXOs, miR-185-5p had the largest readcounts (Figure 5B). Furthermore, miR-31a-5p and miR-185-5p were the two with the largest fold change (Figure 5C). Hence, miR-185-5p was selected for following studies. The small RNA sequencing results were corroborated *via* RT-qPCR which demonstrated a significant decrease in the levels of miR-185-5p in APP-EXO versus EXO (Figure 5D). To make sure that our observations *in vitro* match with the situation *in vivo*, we examined the exosomal miR-185-5p levels in AD mouse brains *via* RT-qPCR and found a significant decline of miR-185-5p expression levels in AD-EXO compared with Ctl-EXO (Figure 5E).

Next, we carried out dual-luciferase assay to validate the direct binding of miR-185-5p with *AP*P 3′UTR (Figure 5F). Co-transfection of miR-185-5p mimics and dual-luciferase reporter constructs containing the wild-type APP 3′UTR, but not miR-185-5p target site-mutated *APP* 3′UTR, significantly decreased the firefly activity in N2a cells, normalized by the Rellina activity, suggesting that miR-185-5p directly targets *APP* transcripts. We further tested that whether or not the sorting miR-185-5p in exosomes is regulated by *APP* 3′UTR. RT-qPCR results showed that miR-185-5p levels was significantly reduced in UTR-EXO, but not in CDS-EXO, compared with EXO, indicating *APP* 3′UTR but not CDS as the key element in regulating the sorting of miR-185-5p into exosomes (Figure 5G). Taken together, our study identified miR-185-5p as an EXO-enriched miRNA whose loading into exosomes is greatly influenced by endogenous *APP* 3′UTR.

### Neuronal Exosomes With Low miR-185-5p Levels Promote APP Expression in Recipient Cells

**FIGURE 6:**
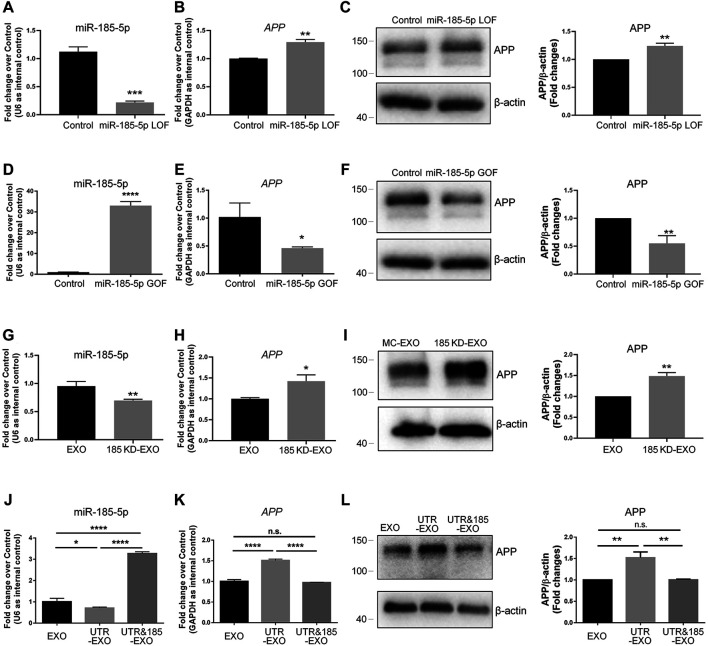
Exosomal miR-185-5p is important to inhibit APP expression in recipient cells. **(A)** N2a cells were transfected with either scrambled control or miR-185-5p inhibitor for 2 days. The expression levels of miR-185-5p were determined by RT-qPCR. **(B)** The up-regulation of *APP* transcript expression in miR-185-5p inhibitor-transfected N2a cells were determined by RT-qPCR. **(C)** The up-regulation of APP protein expression in miR-185-5p inhibitor-transfected N2A cells were determined by western blotting. **(D)** N2a cells were transfected with either scrambled control or miR-185-5p mimics for 2 days. The expression levels of miR-185-5p were determined by RT-qPCR. **(E)** The down-regulation of *APP* transcript expression in miR-185-5p mimics-transfected N2a cells were determined by RT-qPCR. **(F)** The down-regulation of APP protein expression in miR-185-5p mimics-transfected N2a cells were determined by western blotting. **(G)** Exosomes were collected from the conditioned medium of N2a cells transfected with either inhibitor control or miR-185-5p inhibitor. The expression levels of exosomal miR-185-5p were determined by RT-qPCR. **(H,I)** N2a cells were co-cultured with either EXO or 185 KD-EXO for 2 days. The expression levels of *APP* transcripts and APP protein in exosome-co-cultured N2a cells were determined by RT-qPCR **(H)** and western blotting **(I)**. **(J)** Exosomes were collected from the conditioned medium of N2a cells co-transfected with either empty vector + mimics control, *APP* 3′UTR + mimics control, or *APP* 3′UTR + miR-185-5p mimics. The expression levels of exosomal miR-185-5p were determined by RT-qPCR. **(K,L)** N2a cells were co-cultured with either EXO, UTR-EXO, or UTR&185-EXO for 2 days. The expression levels of *APP* transcripts and APP protein in exosome-co-cultured N2a cells were determined by RT-qPCR **(K)** and western blotting **(L)**. Data were represented as mean ± s. d. from three independent experiments. *, **, ***, and **** denote *p* < 0.05, *p* < 0.01, *p* < 0.001, and *p* < 0.0001, respectively.

To determine whether miR-185-5p mediates the effects of APP-EXO, we first examined the roles of miR-185-5p in regulating APP expression in neuronal cells *via* miR-185-5p loss-of-function (LOF) and gain-of-function (GOF) approaches. For miR-185-5p LOF, we transfected N2a cells with either miR-185-5p inhibitor (=LOF group) or inhibitor control for 2 days. RT-qPCR analysis showed a significant decrease in the levels of miR-185-5p in miR-185-5p LOF group, validating the efficiency of transfection (Figure 6A). A significant increase of *APP* transcript levels was observed in LOF group, compared with controls (Figure 6B). RT-qPCR results were confirmed by western blotting, which showed a significant increase of APP protein levels in the cell lysate of miR-185-5p LOF group versus that of control group (Figure 6C). For miR-185-5p GOF, N2a cells were transfected with either miR-185-5p mimics (=GOF group) or mimics control for 2 days. The efficiency of transfection is validated by RT-qPCR (Figure 6D). Moreover, both RT-qPCR and western blotting analyses demonstrated a significant decline of the expression levels of *APP* transcripts (Figure 6E) and APP protein (Figure 6F), respectively, in miR-185-5p GOF group, compared with control group. Thus, our results suggested that miR-185-5p negatively regulated *APP* expression in neuronal cells *in vitro*.

Afterwards, we transfected N2a cells with miR-185-5p inhibitor to knockdown (KD) intracellular miR-185-5p and collected exosomes from the conditioned medium of miR-185-5p KD cells (185 KD-EXO). The KD of exosomal miR-185-5p was validated by RT-qPCR (Figure 6G). N2a cells were co-cultured with either EXO control or 185 KD-EXO for 2 days. RT-qPCR results showed a significant increase of *APP* transcripts in 185 KD-EXO treated cells versus controls (Figure 6H). The RT-qPCR data were further confirmed by western blotting (Figure 6I), indicating miR-185-5p as an essential content of exosomes for inhibiting APP expression in recipient cells.

Since the small RNA sequencing analysis revealed multiple miRNAs whose abundance in exosomes were regulated by *APP* 3′UTR, we next addressed that whether exosomal miR-185-5p mediates the promoting effects of UTR-EXO on APP expression. Except for EXO and UTR-EXO, we also collected exosomes from the conditioned medium of N2a cells co-transfected with *APP* 3′UTR and miR-185-5p mimics (UTR&185-EXO). RT-qPCR results revealed that the *APP* 3′UTR overexpression-induced decline of the levels of exosomal miR-185-5p was abrogated by miR-185-5p GOF in exosome-secreting cells (Figure 6J). N2a cells were then co-cultured with either EXO, UTR-EXO, or UTR&185-EXO for 2 days. RT-qPCR analysis found no significant difference of APP transcripts in UTR&185-EXO group, compared to control group, which indicated that the UTR-EXO-induced excessive expression of *APP* transcripts in recipient cells was rescued by the overexpression of exosomal miR-185-5p (Figure 6K). Western blotting results also displayed a similar APP protein expression pattern to that of RT-qPCR (Figure 6L). In conclusion, our findings suggest miR-185-5p as an important exosomal cargo in modulating APP expression in recipient cells, which may contribute to the dysregulation of APP metabolism in AD.

### miR-185-5p Levels Are Down-Regulated in Exosomes Derived From AD Patients’ and Mouse Sera

**FIGURE 7:**
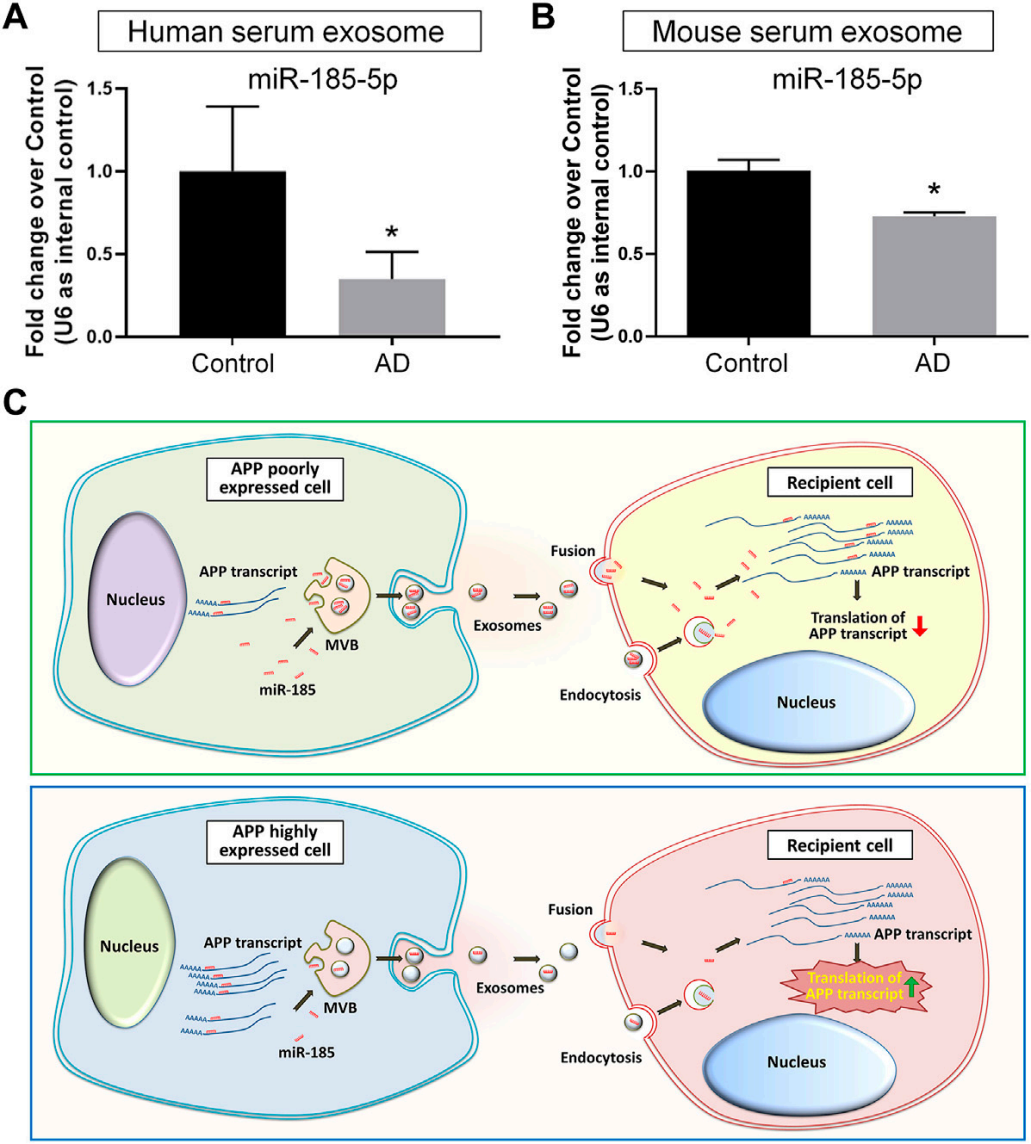
Serum exosomal miR-185-5p is lower in AD patients and mice than healthy controls. **(A)** Human serum exosomes were isolated from three AD patients and three healthy donors. The levels of miR-185-5p in exosomes were determined by RT-qPCR. **(B)** Mouse serum exosomes were isolated from three APP/PS1 mice and three control mice. The levels of miR-185-5p in exosomes were determined by RT-qPCR. **(C)** A schematic representation of exosome-mediated APP regulation. Upper panel: in normal conditions, the low levels of endogenous APP transcripts in normal neuronal cells result in high levels of free intracellular miR-185-5p. Consequently, more miR-185-5p can be loaded into exosomes and delivered to recipient cells *via* exosomes for maintaining APP expression at a low level in recipient cells. Lower panel: in pathological conditions, the expression levels of APP transcripts are abnormally elevated in neuronal cells. Excessive APP transcripts bind to more miR-185-5p, reducing free miR-185-5p intracellularly and leading to less miR-185-5p that can be loaded into exosomes. Exosomes with less miR-185-5p fail to suppress APP expression in recipient cells, causing uncontrolled APP expression. Data were represented as mean ± s. d. * denotes *p* < 0.05.

As our *in vitro* observations indicated exosomal miR-185-5p as a key regulator of APP expression that may be involved in AD pathogenesis, we examined the potential translational value of exosomal miR-185-5p in AD diagnosis. We first compared the exosomal miR-185-5p levels in the sera collected from three AD patients and three age-/gender-matched healthy donors. RT-qPCR results suggested that serum exosomal miR-185-5p was significantly down-regulated in AD group versus controls (Figure 7A). We also collected sera from three AD mice and three age-/sex-matched C57 mice. Similarly, RT-qPCR results showed that the expression levels of serum exosomal miR-185-5p were significantly lower in AD mice, compared with controls (Figure 7B). Hence, our results implied a possibility of utilizing serum exosomal miR-185-5p in AD diagnosis, which requires extensive investigations including the introduction of large AD cohort and complicated statistical analyses.

## Discussion

With the rapid expansion of our knowledge, exosomes have now been considered as a key contributor in the pathogenesis of AD (Malm et al., 2016; Xia et al., 2019b). The inhibition of exosome release has obtained outstanding therapeutic effects including the suppression of glial activation, the reduction of total Aβ_42_ and plaque burden, the down-regulation of tau phosphorylation, and the enhancement of cognitive functions in animal studies (Dinkins et al., 2014; Dinkins et al., 2016). Since exosomes mediate various physiological processes, the shutdown of exosome biogenesis may also induce adverse reactions such as the impairment of anti-inflammatory responses and cell survival in the CNS (Chen et al., 2019; Yue et al., 2019). Therefore, more attentions have been paid on the identification of AD-related cargos in exosomes. For example, Aβ_42_ has been identified in neuronal exosomes isolated from AD patients’ peripheral blood and cerebrospinal fluid (Jia et al., 2019). Similarly, hypo-phosphorylated Tau protein can be detected in exosomes derived from primary cortical neurons (Wang et al., 2017). The transmission of exosomal cargos among neurons therefore acts as an essential channel for the spreading of Aβ and Tau in the brain (Jiang et al., 2019). Since current theories deem the formation of Aβ senile plaques and Tau neurofibrillary tangles as clinico-pathological features of AD instead of the causes of AD (Armstrong, 2013), the crosstalk between exosomes and AD-related genes including APP, presenilin (PSEN), and apolipoprotein E (ApoE) has been given with more and more emphasis (Perez-Gonzalez et al., 2012; Fernandes et al., 2018; Fyfe, 2019; Peng et al., 2019). In this study, we have demonstrated that exosomes derived from N2a cells with APP ectopic expression significantly increase the expression of APP in recipient cells. These exosomes achieve their functions highly likely through the delivery of miR-185-5p, instead of the direct transferring of *APP* gene products to recipient cells. Our study indicated that healthy neurons-derived exosomes, containing high levels of APP inhibiting factors including miR-185-5p, act as a cell-extrinsic sustainer for the inhibitory networks of APP expression. The dysregulation of APP expression confines miR-185-5p to APP 3′UTR, leading to the collapse of the inhibitory networks (Figure 7C).

To date, miR-185-5p has emerged as a key regulator of the pathogenesis of various diseases including tumor (Niu and Tang, 2019). miR-185-5p targets *ROCK2* (Niu and Tang, 2019), *ELK1* (Fan et al., 2018), *ELK3* (Liu et al., 2020), *IGF2* (Zhuang et al., 2020), *RAGE* (Yin et al., 2018), *BCL2*, *BCL2L1* (Ostadrahimi et al., 2018), and many other genes to suppress tumor cell migration and invasion. miR-185-5p dysregulation has also been linked to renal fibrosis (Yuan et al., 2020), intervertebral disc degeneration (Guo et al., 2020), and other diseases (Li et al., 2019). A significant decrease in the levels of serum exosomal miR-185-5p in AD patients has been reported in a comparative study (Lugli et al., 2015). Here, we confirmed this pattern using samples collected from both AD patients and animal models. More importantly, our study is the first one showing the direct interaction between miR-185-5p and APP 3′UTR, which controls the sorting of miR-185-5p into exosomes. Our observations match with the findings of Squadrito et al. that endogenous mRNA down-regulates its targeting miRNAs sorting into exosomes *via* anchoring free miRNAs to its 3′UTR (Squadrito et al., 2014). Hence, we, in this study, demonstrated the tight association between the abnormal expression of AD-related genes and the shift of exosomal miRNA patterns in the patients’ biological fluids.

Mounting evidence has implicated exosomal miRNAs as biomarkers of AD. Lugli et al. found that plasma exosomal miR-185-5p, together with other six miRNAs, was sufficient to allow highly accurate prediction of AD in individual samples (Lugli et al., 2015). But whether plasma/serum exosomal miR-185-5p alone can be used as an AD biomarker remains unknown. In this study, although we identified a significant reduction in the levels of exosomal miR-185-5p in patients’ serum, the small sample size did not allow us to evaluate the sensitivity and specificity of exosomal miR-185-5p in AD diagnosis. With a larger sample size and more extensive investigations, the potential application of serum exosomal miR-185-5p in the diagnosis of AD may be a near possibility.

In summary, our study has demonstrated that the excessive expression of neuronal APP reduces exosomal miR-185-5p levels through the direct binding of *APP* 3′UTR to miR-185-5p, therefore releasing *APP* transcripts in the exosome-receiving cells from exosomal miR-185-5p-mediated translation inhibition. Thus, our study provides a possible mechanism for the intercellular regulation of APP expression *via* exosomes, implying exosomes and their miRNAs as potential therapeutic targets for AD treatment and biomarkers for AD diagnosis.

## Data Availability

The datasets presented in this study can be found in online repositories. The names of the repository/repositories and accession number(s) can be found below: ArrayExpress: The accession number: E-MTAB-11106.

## Abbreviations

185 KD-EXO, miR-185-5p-knockdown N2a cell-derived exosomes; AD, Alzheimer’s disease; abrAPP-N2A, N2a cells with APP overexpression; ApoE, Apolipoprotein E; APP-EXO, APP-overexpressed N2a cell-derived exosomes; Aβ, Amyloid-β; CDS: Coding DNA sequence; CDS-EXO, APP coding DNA sequence-overexpressed N2a cell-derived exosomes; CNS, Central nervous system; EXO, N2a cell-derived exosomes; GO, Gene ontology; GOF, Gain-of-function; KD, Knockdown; KEGG, Kyoto Encyclopedia of Genes and Genomes; LOF, Loss-of-function; MCI, Mild-cognitive impairment; miRNA, microRNA; mRNA, Messenger RNA; NTA: Nanoparticle tracking; OE, Overexpress; PD, Parkinson’s disease; PSEN, Presenilin; UTR, Untranslated region; UTR-EXO, APP 3′untranslated region-overexpressed N2a cell-derived exosomes.
